# Proof of Concept for the Organic Electrorefinery Technology with Actual Effluents

**DOI:** 10.1021/acs.iecr.4c02235

**Published:** 2024-10-22

**Authors:** Jesús Parrilla, Inalmar Dantas Barbosa Segundo, Carmen María Fernández Marchante, Elisama Vieira Dos Santos, Justo Lobato, Suely S. L. Castro, Carlos Alberto Martínez-Huitle, Manuel Andrés Rodrigo

**Affiliations:** † Chemical Engineering Department, University of Castilla-La Mancha, Ed. Enrique Costa Novella, Campus Universitario s/n, Ciudad Real 13005, Spain; ‡ School of Science and Technology, Federal University of Rio Grande do Norte, Campus Universitário, Av.Salgado Filho 3000, Lagoa Nova, Natal, RN CEP 59078-970, Brazil; § Renewable Energies and Environmental Sustainability Research Group, Institute of Chemistry, Federal University of Rio Grande do Norte, Campus Universitário, Av. Salgado Filho 3000, Lagoa Nova, Natal, Rio Grande do Norte CEP 59078-970, Brazil; ∥ Faculty of Exact and Natural Sciences, State University of Rio Grande do Norte, Campus Central, Mossoró, Rio Grande do Norte P59625-620, Brazil

## Abstract

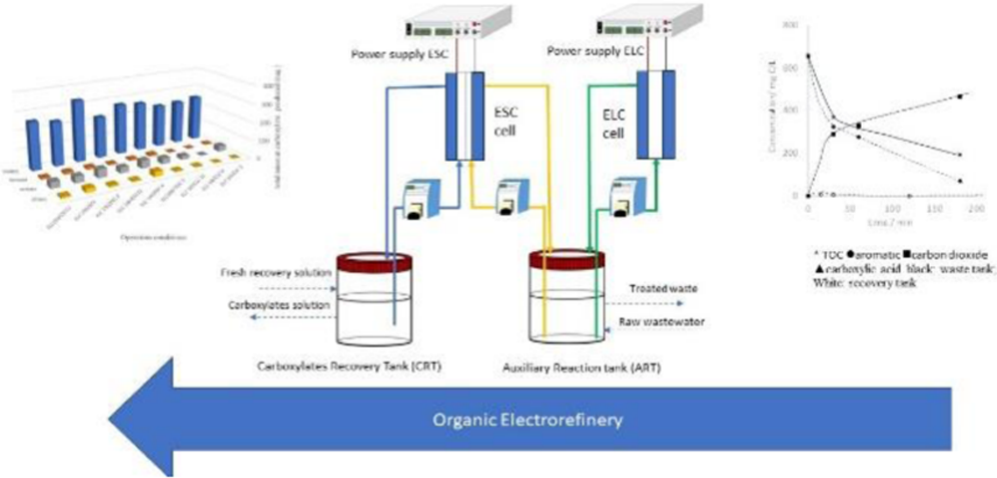

This work describes results of a first proof of the concept of electrorefinery with a real waste obtained from a cashew nut factory, and it shows the effect of the current densities of both the anodic oxidation and electrochemically assisted separation processes on the performance of the system. Results obtained demonstrate that electrorefinery is a promising option to minimize the carbon fingerprint, worth studying for increasing the sustainability of the environmental remediation of wastes, because valuable species can be obtained from the destruction of pollutants and recovered within the same integrated process. They also point out that there is still a long way to reach an optimum solution for this technology, but it is worth the effort to be made. Many different carboxylates were detected, but oxalate was the primary product both in the reaction tank and in the recovery tank. The production is almost linear during the electrolysis, with a reaction rate of 23.3 mg C h^–1^ in the case of oxalate and a separation ration of around 20% in the electrodialysis stage. There is a negligible crossover of aromatic species into the recovery solution, which becomes an important advantage for further processing of the carboxylate solutions in the search to valorize these species in terms of circular economy principles. Energy efficiencies in the range of 0.04–0.21 mg C-carboxylates (Wh)^−1^ and Coulombic efficiencies in the range 0.92–2.03 mg C-carboxylates (Ah)^−1^ were obtained in this work. A life cycle assessment indicated carbon dioxide and water footprints as low as 0.31 g of CO_2_ mg^–1^ C and 30 mL of H_2_O mg^–1^ C recovered in the products obtained, respectively.

## Introduction

Nowadays, there is a growing concern related to the generation and treatment of wastes. Society is aware that it is not enough to treat wastes, as it was the environmental paradigm decades ago, and that it is necessary to go further on this treatment objectives, looking for reaching a more sustainable solution, which helps to mitigate the important environmental problems that are arising on Earth, including the global warming and the exhaustion of natural resources.^1,2^

Due to the fact that the world’s population is expected to reach 8 billion people by 2022 (UNFPA, 2023) and is predicted to continue growing, it is imperative to make wise use of natural resources while protecting the environment, taking into account the life cycles of living beings and the planet’s resources. For this reason, the study of socio-environmental impacts, life cycle assessments, or exergoenvironmental analysis is becoming more and more important when making decisions that have an impact on human development.^3−5^ Thus, modern society is on the way to change the current waste treatment concept by the paradigm of circular economy^6−8^ in which waste is used to recover raw matter avoiding their exhaustion from Earth and contributing positively to the decrease in the carbon and water fingerprint of both, production and environmental technologies.^9^ A good starting point in this approach was the concept of biorefinery, which emerged in the 1990s with the goal to valorize biomass resources in an economical and environmental way by mimicking the well-developed concept of a petroleum refinery,^10^ where oil is refined into many marketable products including chemicals, energy, and fuels,^11^ using different types of biomass as renewable feedstock materials, instead of nonrenewable fossil fuels oil.^12^

In recent times, attempts have been made to go a step further by trying to exploit new strategies in different fields.^13^ Electrochemical engineering is trying to develop its own view by facing the modification of the target of environmental electrochemical technology from the destruction of pollutant to a new concept called electrorefinery in organics.^14^ Thus, electrolytic technology has been found to be a suitable alternative to other treatments in the degradation of organic pollutant contained in not only in liquid and gaseous streams but also in soil remediation.^15^ With the development of diamond or tailored mixed metal oxides coatings, efficiencies reached by electrolytic technologies are competitive to those of other technologies.^16,17^ However, the target has been up to now the mineralization, that is, the transformation of organic pollutants into carbon dioxide, focusing mainly on anodic oxidation reactions.^18^ This approach seems to be contradictory with the fact that one of the most interesting research topics currently tackled in electrochemistry is the transformation of carbon dioxide into valuable products using cathodic reduction reactions that aims to produce carboxylates or alcohols.^19^ At this point, the extensive knowledge produced during the last three decades allows to know that in the transformation of complex organics to carbon dioxide, there are a sequence of oxidation processes, which involves the progressive transformation of functional groups of the organic pollutants, first to alcohols by the addition of hydroxyl radicals, then to aldehydes and ketones by the oxidation of the alcohols and finally to carboxylic acids and/or with the breakage of the molecules.^20^ The hazardousness of the waste is typically reduced during this progression, as well of the added values of the products formed^21^ and exergy of organic molecules.

Based on the existing literature, few works have been published considering real biomass effluents to produce high value-added products, like carboxylic acid and carboxylates. As can be observed in Table 1, the electrochemical conversion of real biomass effluents promotes the production of carboxylic acids and carboxylates, but their accumulation was not the scope of these investigations.

**Table 1:** Selected Examples of Electrochemical Conversion of Biomass Real Wastewaters into Carboxylic Acids/Carboxylates as High Value-Added Products

	**concentration** (mg/L)				
**carboxylic acid/carboxylates**	**Di Marino et al**. (2019)	**Medeiros et al**. (2020)	**Medeiros et al**. (2022)	**Oliveira et al**. (2023)	**Brix et al**. (2021)
acetic	210.0^a^	309.0^c^	63.5^f^	45.7^i^	24.02^k^
formic	1340.0^a^	7.0^e^	2.0^g^	7.6^j^	17031.1^k^
fumaric		1.0^e^	<1.0^f^^,^^g^^,^^h^		
malic	8.5^a^	0.45^d^	1.5^h^		
malonic	19.0^b^				
oxalic	513.0^b^	630.0^c^	4.1^g^	4.9^i^	1125.4^k^
succinic	15.5^b^				
tartaric					
glyceric					487.97^k^
glycolic					2129.4^k^
lactic					3242.88^k^
tartronic					60.03^k^

a 100 mL of 5 g/L of kraft lignin with 1 mol/L NaOH, 2.5 V, 7 h, divided cell by a polymer spacer, nickel foam (5.624 cm2) as working and counter electrodes.

b 100 mL of 5 g/L of kraft lignin with 1 mol/L NaOH, 3.5 V, 7 h, divided cell by a polymer spacer, nickel foam (5.624 cm2) as working and counter electrodes.

c 250 mL of 0.1% t-CNSL in 1.00 mol/L NaOH, 70 mA cm^–2^, 180 min, undivided batch cell, Nb/BDD (10 cm2) as anode and Ti (10 cm2) as cathode.

d 250 mL of 0.1% t-CNSL in 1.00 mol/L NaOH, 70 mA cm^–2^, 60 min, undivided batch cell, 250 mL, Nb/BDD (10 cm2) as anode and Ti (10 cm2) as cathode.

e 250 mL of 0.1% t-CNSL in 1.00 mol/L NaOH, 100 mA cm^–2^, 180 min, undivided batch cell, Nb/BDD (10 cm2) as anode and Ti (10 cm2) as cathode.

f 250 mL of 0.01% of t-CNSL in 1 mol/L NaOH, 70 mA cm^–2^, 30 min, undivided batch cell, DSA (Ti/TiO_2_RuO_2_IrO_2_, 10 cm2) as anode and Ti (10 cm2) as cathode.

g 250 mL of 0.01% of t-CNSL in 2 mol/L NaOH, 40 mA cm^–2^, 30 min, undivided batch cell, DSA (Ti/TiO_2_RuO_2_IrO_2_, 10 cm2) as anode and Ti (10 cm2) as cathode.

h 250 mL of 0.01% of t-CNSL in 1 mol/L NaOH, 100 mA cm^–2^, 30 min, undivided batch cell, DSA (Ti/TiO_2_RuO_2_IrO_2_, 10 cm2) as anode and Ti (10 cm2) as cathode.

i 1000 mL of a real effluent of washing machine with 0.1 mol/L Na2SO4, 60 mA cm^–2^, 150 min, PEM divided cell membrane, Nb/BDD (15 cm2) as anode and Ni–Fe based SS mesh (18.2 cm2) as cathode.

j 1000 mL of a real effluent of washing machine with 0.1 mol/L Na2SO4, 7.5 mA cm^–2^, 150 min, PEM divided cell, Nb/BDD (15 cm2) as anode and Ni–Fe based SS mesh (18.2 cm2) as cathode.

k 12.5 mL of effluent of 0.5 mol L^–1^ glycerol, 2 mol L^–1^ KOH, *j* = 2.5 mA cm^–2^, 1440 min. Ni-boride(1 cm^2^), Ni-boride (1 cm^2^), anion exchange membrane.

As already discussed in several investigations in which the degradation of pollutants is the main scope, several carboxylic acids are produced at the end of the electrochemical treatments.^14^ These results have indicated that the nature of the anodic material and experimental factors (hard oxidation conditions) can influence the distribution and accumulation of carboxylic acids produced. Nevertheless, the recent investigations related to the electrochemical treatment of effluents are fulfilled with the principles of circular economy by obtaining valuable compound acids.^14^ For example, evolution of the carboxylic acids (acetic, formic, malic, malonic, oxalic, and succinic acids) was attained when 100 mL of 5 g L^–1^ of kraft lignin in 1 mol L^–1^ NaOH was electrolyzed by applying 142 mA m^–2^ during 420 min.^22^ However, the accumulation of acetic (210.0 mg L^–1^) and formic (1340 mg L^–1^) acids was selectively electrogenerated in alkaline conditions, in an undivided reactor (swiss-roll cell) by using a Ni foam electrode (see Table 1). Other example is the electrolysis of glycerol^23^ with Ni-boride electrodes using a divided cell under alkaline and soft oxidation conditions (12.5 mL of effluent of 0.5 mol L^–1^ of glycerol in 2 mol L^–1^ KOH, *j* = 2.5 mA cm^–2^), significantly improving the accumulation of formic (17031.10 mg L^–1^), glyceric (487.97 mg L^–1^), glycolic (2129.40 mg L^–1^), and oxalic (1125.37 mg L^–1^) acids, even when acetic and tartronic acids are also produced. Taking into consideration the results obtained by other authors,^14,23−27^ the investigation about of the optimal operating parameters to promote the selective production of carboxylic acids from the electrochemical conversion of dissolved organic matter in a biomass effluent was the main objective of our previous works.^28,29^ Then, the production of acetic, formic, and oxalic acids was favored when 0.1% t-CNSL effluent was electrolyzed (at 40 mA cm^–2^) with the BDD anode in an alkaline medium (1.0 mol L^–1^ NaOH),^28^ reaching an accumulation of about 144, 120, and 75 mg L^–1^, respectively, after 240 min. However, these concentrations were significantly boosted by increasing the *j*, achieving 630 mg L^–1^ for oxalic acid by applying 70 or 100 mg L^–1^ during 240 min while the concentration of acetic acid significantly increased to 309 and 281 mg L^–1^, for 70 and 100 mA cm^–2^, respectively, during the first 180 min of electrolysis.^28^ In another study, a notable improvement in the selectivity of the conversion of t-CNSL into acetic acid was assessed by the Ti/TiO_2_RuO_2_IrO_2_ anode (0.01% t-CNSL effluent was electrolyzed at 70 mA cm^–2^ in 1.0 mol L^–1^ NaOH), reaching 63.5 mg L^–1^, while oxalic acid was slightly accumulated (4.1 mg L^–1^).^29^ It is important to note that in both studies, a batch reactor with a volume of 250 mL was used, but a lower concentration of biomass was electroconverted, in the latter. Also, it is possible to infer that the lower efficiency can be affected by the simultaneous evolution of the oxidation and reduction reactions in the t-CNSL conversion. Therefore, the “electrorefinery for organics” concept can be extended to other renewable sources, not limited to biomass, such as glycerol^30^ and wastewaters.^31,32^

In light of the discussions above, these results allowed to understand that the valorization of the wastes is feasible, highlighting the importance of the electrochemical reactor design and the selection of the nondestructive operation conditions to selectively enhance the concentration of the organic acids.^14^ However, it is important to remark that the concept is slightly different than a biorefinery where the microorganisms are the protagonist of the chemical reactions to convert from pollutants to new compounds, and the separation of valuable products is still a handicap because what is produced is a mixture of the raw pollutants with reaction intermediates and the cost of purification of any intermediate may seem very often to be unaffordable nowadays.

Within this frame, an efficient separation will open the possibility to explore new electrosynthesis or chemical organic synthesis procedures capable of transforming these intermediate species into a valuable product, that is, integrating electrochemical technologies into the new circular economy paradigm by using these species as “bricks”.^33^ On the other hand, purification of the species is a topic of the major interest at this point, to provide a suitable feedstock for these processes. However, refractory characteristics of carboxylic acids (specially of those with a short chain) can help to identify these species as the target of the treatment, because although the oxidation is sequential, they seem to be the more easily accumulable species.^34−36^ One important characteristic of these species is that they can be ionized (deprotonated) by changing the pH of the solution where they are contained, and this will be very effective if the change is far away from the p*K*_a_ of the pair carboxylic acid/carboxylate.^37^ This opens the possibility of process integration in which the electrochemical treatment will be transformed into a refinery of wastes as feedstock to produce carboxylates.

In this work, it is going to propose and test a new simple prototype of electrochemically assisted refinery (or simply electrorefinery) based on the integration of two electrochemical stages into the same proof of concept plant: waste electrolysis and carboxylate electrochemical separation using anionic membranes. This new approach is going to be applied to a real waste, produced during the processing of cashew nut (*Anacardium occidentale* L.), which is a tropical nut tree that provides the cashew fruit. The tropical tree (*Anacardium occidentale* L.) is native from South America,^38,39^ but now, it has been expanded to more than 33 tropical countries. It was initially planted to improve soil properties and prevent erosion in tropical coastal areas, although its expansion has increased significantly due to the growing demand for this type of nut in the global population’s diet, which has been influenced by the rise in vegan diets (Catarino et al., 2015). Thus, there was an increase in the global cashew acreage between 1980 and 2020 from 5262.50 to 71,019.7 km^2^^5,40^ and various of its components (e.g., kernel) are valued not only and food proposes but even also for medicines.

The cashew fruit has the peculiarity of consisting of two edible parts: accessory fruit, also known as the pedicel, and true fruit, which is the cashew nut encased in a hard shell. Processing of the raw nut results in approximately 67% cashew nut shells and a cashew nutshell liquid (CNSL). As a byproduct of cashew nut processing, cashew nutshell liquid is extracted from the nutshell and makes up approximately 20–25% by weight of the nut.^28,29,41−43^ The cashew nutshell liquid has three main components, anacardic acid, cardanol, and cardol, being classified as natural (n-CNSL) or technical (t-CNSL), depending on the extraction method; the n-CNSL is extracted with solvents, while the t-CNSL is obtained burning the nuts industrially at high temperatures. Its unique structural features (phenolic hydroxyl, aromatic ring, and unsaturation(s) in the alkenyl side chain), abundant availability, and low cost make this byproduct extensively used for industrial applications, such as, biodiesel, oil and gas industries, adhesives, and surfactants.^43,44^ For these reasons, CNSL has a significant commercial value.^45^ An estimated 2.5 million tons of cashew nuts are produced annually worldwide. Only around 25% of the cashew nut shells are really used; the remainder are burned outdoors. This kind of treatment produces a lot of waste and emissions of greenhouse gases^43^ and when it rains, water washes some of the acidic oil from the cashew nut into rivers and reservoirs, putting people and the environment at risk. Currently, this industry generates a large amount of waste that is not properly managed, so it would be of great interest to recover this waste, as proposed in refs (28) and (29). In relation to the above discussion, electricity emerges as an effective technique for the valorization of CNSL, to obtain high value-added products from it. Moreover, the sustainability of these processes can even be improved with the use of renewable energy sources. In this context, although the TRL of the proof of concept proposed in this work is low, this paradigm change is extremely important to improving sustainability in the production chain of any organic product. In particular, improving the technical, economic, and environmental feasibility of the cashew nut production process by integrating electrochemical technology is a new challenge that can serve as a reference for other organic products with the aim of maximizing the use of the biosphere’s resources.

### Description of the Proof of Concept

**Figure 1:**
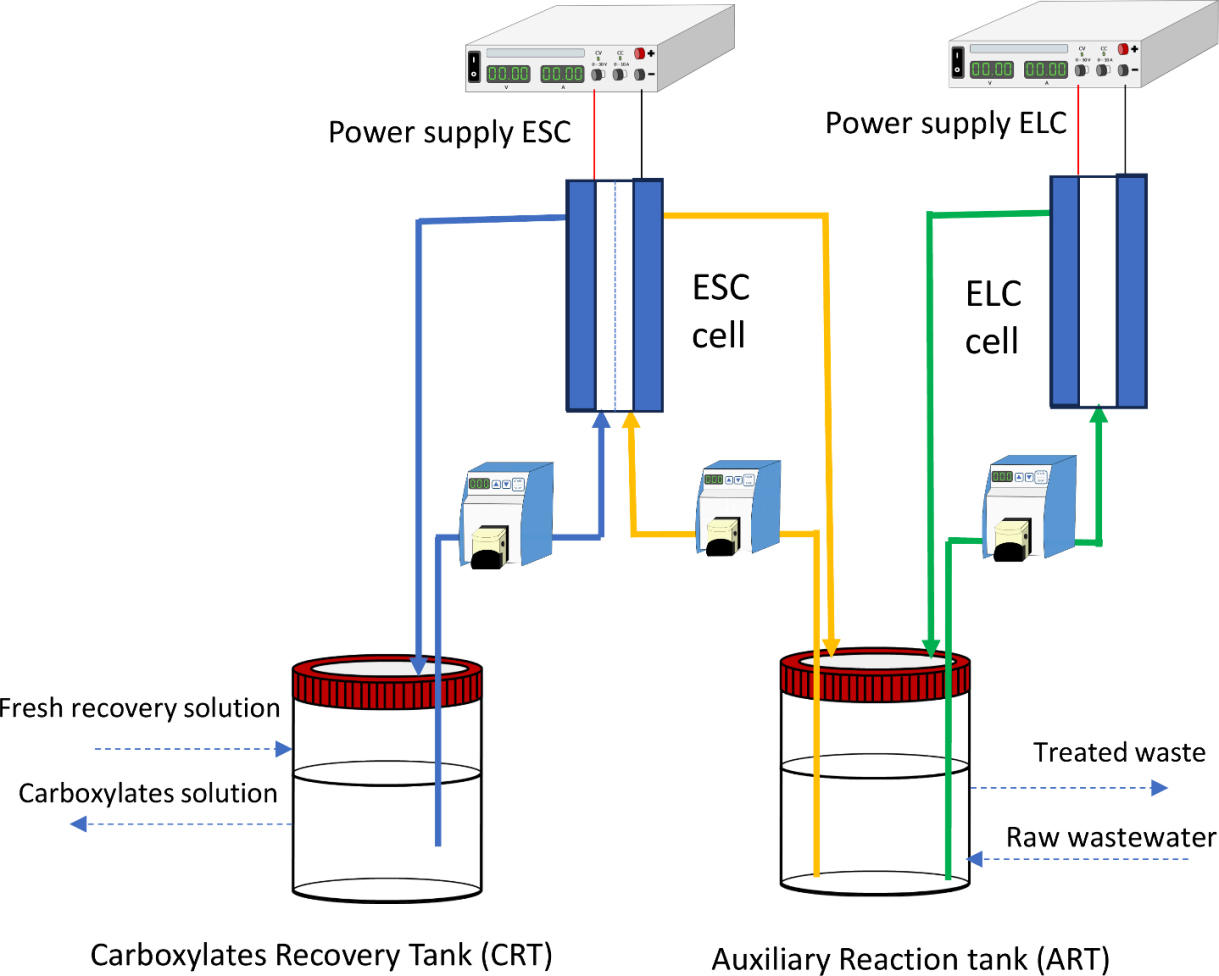
Scheme of the proof of concept, including the electrochemical cell installations and connections between the reservoirs.

A scheme of the process proposed to develop the electrorefinery concept is shown in Figure 1.

The electrolysis in a single compartment electrochemical cell allows the sequential transformation of complex organics into more simple species, and before it ends up in the production of carbon dioxide (mineralization), the last stage is the formation of carboxylic acids. These acids can be easily deprotonated by operating at pH ranges above their p*K*_a_, and they form carboxylates.^37^ Stopping the reaction in this stage is not possible now, because there are no electrodes tailored to oxidize organics up to this point and they become inefficient for this last stage of mineralization. However, an appropriate combination of the operation current density (and, subsequently, of the applied cell voltage) and the choice of suitable anode coatings may help to increase the selectivity of the reaction toward carboxylates.

The electrodialysis allows for the transport of charged species through membranes by electromigration, a transport mechanism that depends on the electric field produced in an electrochemical cell. In the simplest case, an electrochemical cell equipped with an anionic exchange membrane, which is separated by an anodic and cathodic compartment (so-called electroseparation cell or ESC in this work), can be used to concentrate these carboxylates into the anodic compartment if a concentrated solution containing the carboxylates (such as the in-treatment waste) is passed through the cathodic compartment. Efficiency of this process can be easily improved by enhancing the design of the ESC using other configuration of electrodialysis cell with a larger number of compartments, but for a proof of concept, this simple design may be considered as suitable. Here, it is also important to select operation current densities that do not promote the oxidation of the carboxylates on the anode or their reduction of the cathodes and, as well, electrodes that promote water oxidation and reduction instead of organic reaction to promote their accumulation versus their transformation.

Initially, integration of both processes can be easily obtained (see Figure 1) by connecting the cathodic compartment of the ESC equipped with an anionic membrane and the electrolysis cell (so-called in the work ELC) to the same tank (so-called, auxiliary reaction tank, or ART), where the raw wastewater is contained (and in a continuous operation process the stream of wastewater should be added and removed from this ART tank, as shown by discontinuous arrows) and the cathodic compartment to a tank containing a clean electrolyte solution, where the carboxylates are going to be concentrated (so-called carboxylate recovery tank or CRT). This tank can also be operated in continuous and discontinuous mode as indicated by the discontinuous arrows.

## Materials and Methods

### Reagents

The t-CNSL was collected from a cashew-nut processing industry (Usina Brasileira de Óleos e Castanha, USIBRAS) located in Mossoró/Rio Grande do Norte (Brazil), and it was stored at 20 °C. This material was dissolved in an ethanol solution (2.5 g of resin per 7.5 mL of ethanol), with a subsequent agitation of 20 min. After that, a sample of 0.4 mL of mixture was diluted to 1.0 L of the electrolyte containing 1.0 mol L^–1^ of NaOH (Panreac).^28,29^ Other 20 min agitation took place at this point. Before the experiments were carried out, vacuum filtration was performed with a Büchner funnel to remove some solid particles. This process was very important in the preparation of the initial raw wastewater. All reagents used in this study were of analytical grade, and the solutions were prepared with ultrapure water obtained from a Millipore Milli-Q system with resistivity >18 MΩ at 25 °C.^29^ The pH values of the raw wastewater and the electrolyte used were 12.47 and 11.89, respectively.

### Experimental Setup

The experimental setup fits the proof-of-concept scheme shown in Figure 1. Two power supplies (AD Instruments, model KPS3030E, Spain) were used to power the electroseparation cell (ESC) and the electrolysis cell (ELC). The ELC is a single-pass flow electrochemical cell equipped with a boron-doped diamond (BDD) electrode (Metaken, Germany) and an AISI 314 stainless steel cathode, both with an electrode surface area of 12 cm^2^. The ESC electrochemical cell is a double compartment electrochemical flow cell equipped with a mixed metal oxide (MMO, Ti/RuO_2_IrO_2_TiO_2_) anode and a cathode of stainless steel, both with an electrode surface area of 25 cm^2^. The membrane that separates both compartments was a Neosepta AMX (ASTOM Corporation, Japan) anionic membrane. In the auxiliary reaction tank (ART), 1.0 L of raw wastewater was contained. It was the initial solution described in the section Reagents. Besides, 200 mL of fresh recovery solution (electrolyte containing 1.0 mol L^–1^ of NaOH) was initially in the carboxylate recovery tank (CRT).

Three peristaltic pumps (Heindolph, Germany) were used for the different electrolyte circuits of the experiment device. Flow rates of the electrolyte in the ELC, catholyte in the ESC, and anolyte in the ESC were of 400, 167, and 383 mL min^–1^, respectively. The flow rate in ELC is based on previous experiments.^28,29^ Tests were performed at 20 °C and atmospheric pressure for 180 min. The processes occurred under the galvanostatic regime in both cells. Current densities applied to the ELC were 50, 100, and 150 mA cm^–2^. Current densities applied to the ESC were 2, 6, and 12 mA cm^–2^. Therefore, nine experiments were performed, combining each of the current densities applied to the ELC (50, 100, and 150 mA cm^–2^) with the other three applied to the ESC (2, 6, and 12 mA cm^–2^).

### Analytical Techniques

The concentration of carboxylic acids obtained (acetic, formic, and oxalic) was measured by high-performance liquid chromatography (HPLC) analyses in a JASCO column at 25 °C and 3 MPa. A volume of 20 μL of the sample was injected. The mobile phase with 5 mM of H_2_SO_4_ was eluted at 0.6 mL min^–1^ during 30 min for each test. Retention times were 9.9, 14.5, and 15.8 min for oxalic, formic, and acetic, respectively. These times were determined by calibration curves, which were carried out using samples ranging from 0 to 100 ppm for each of the acids analyzed. From these curves, the different expressions that correlate the chromatographic area to the concentration in parts per million of the acid to be measured were also obtained. The HPLC detection limit is less than 0.1 ppm; lower values are not necessary for this study. All experiments were run in triplicate, and the withdrawn samples were analyzed in triplicate to minimize the experimental error. Deviations between runs were always lower than 5% for all determinations, attaining a high accuracy.

Additionally, the aromatic intermediates from the real effluent electrochemical treatment were evaluated by a gas chromatography coupled to mass spectrometry (GC-MS) Shimadzu QP2010 SE model equipped with a 30 m long RESTEK-RTX-5MS capillary column (0.25 mm film thickness and 0.25 mm internal diameter) and a quadrupole mass detector was used, following the experimental conditions reported by Andrade et al.^46^ as well as Santos et al.^47^ Chemical oxygen demand (COD) was determined using a Velp ECO-16 digester and a Pharo 100 Merck spectrophotometer analyzer, and total organic carbon (TOC) was measured by a Multi N/C 3100 analyzer (Analytik Jena).

To prepare the samples for all of these analyses, different actions were carried out. First, they were filtered and neutralized at 50% v/v with 0.1 M HCl, since the presence of NaOH in the electrolyte of the solution could be quite harmful for the different equipment. Besides, the samples were diluted in Milli-Q water at a ratio of 1:10.

### Sustainability Analysis

The carbon and water footprints of this prototype have been calculated using SimaPro 9.6.0.1 software, Ecoinvent 3 database, and IPCC 2013 GWP 20a V1.03 and AWARE V1.01 methods.

## Results and Discussion

**Figure 2:**
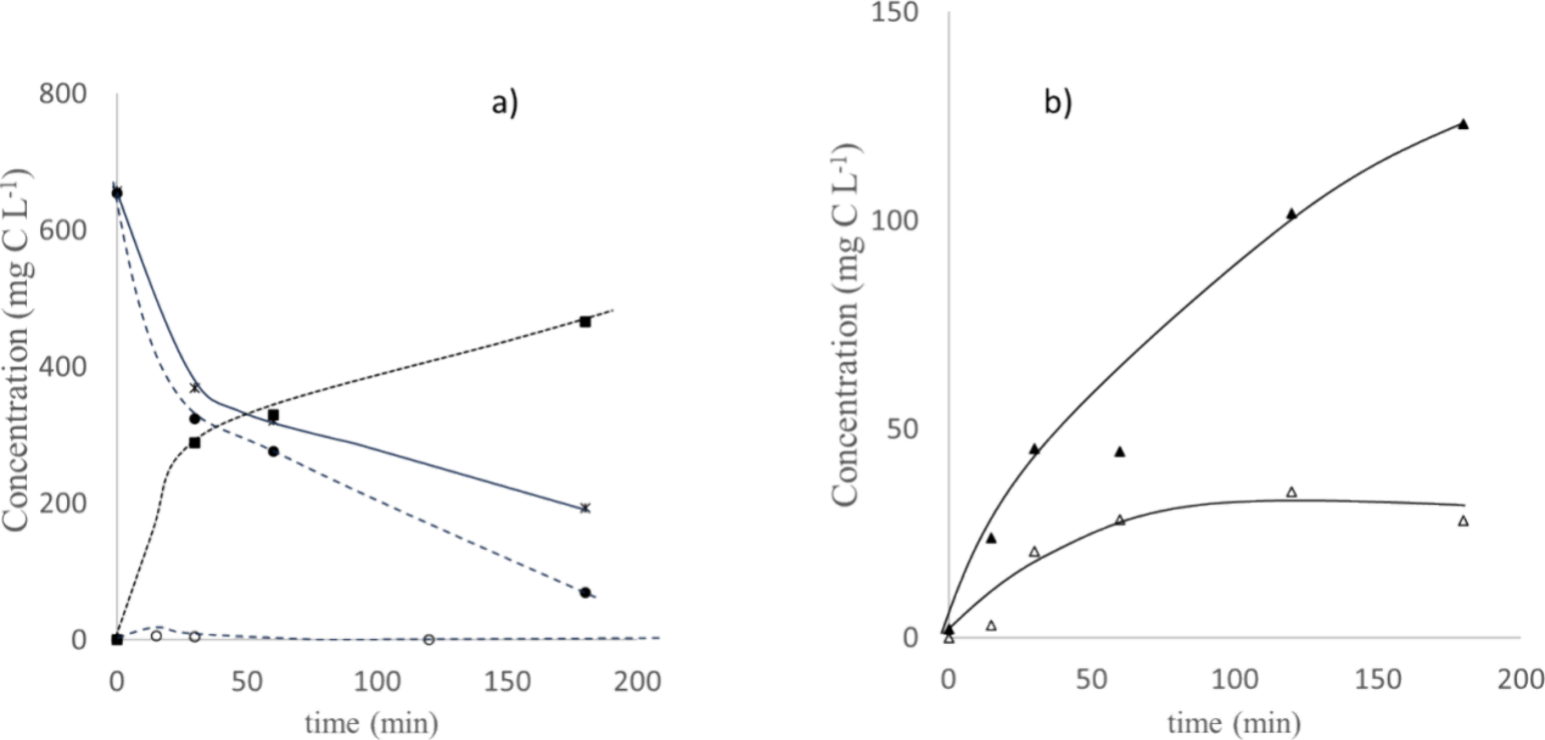
Time course of the main parameters during the application of an electrorefinery test to the real effluent of cashew nut wastewater at 100 mA cm^–2^ (ELC) and 6 mA cm^–2^ (ESC) current densities. Full symbols: waste tank; empty symbols: recovery tank. (a) Total organic carbon (TOC): circle solid, aromatic; box solid, carbon dioxide; (b) triangle up solid, carboxylic acid.

Figure 2 shows the changes observed in the concentration of the main species contained in the two tanks of the electrorefinery proof-of-concept device during the electrolysis of the cashew nut waste in galvanostatic conditions, applying values of current density in both electrochemical reactors, considered as suitable according to literature for anodic oxidation (100 mA cm^–2^) and electrodialysis (6 mA cm^–2^).

As seen, there is an important production of carboxylic acids that are efficiently separated during their production from the waste, being transferred to a clean solution. These results demonstrate the feasibility of the concept of electrochemically assisted refinery with a real wastewater, and they also indicate that it is possible to obtain and, simultaneously, separate valuable products from real wastes, because the carboxylates can be used as precursors of other organic molecules, and they may also have direct applications such as the improvement of production of crops by improving the quality of soils where they are applied.

However, regardless of the integration of the separation with the production of valuable products, it is important to note, according to Figure 2A, that carbon dioxide remains the key product of the electrolysis under these conditions. This is not a surprise, and, in fact, it was an expected result, because the purpose of the proof of concept was to demonstrate that it is possible to split a part of the carboxylates formed during the electrolysis and accumulate them in a different container, not to transform (at the technology readiness level, TRL aimed) all the waste into a valuable product, which will become the target in future developments.^48^

During electrolysis, the chemical oxygen demand (COD) decreases in the ART, because of the oxidation of the pollutants contained in the waste, from 468 down to 164 mg L^–1^ within 3 h of application of current. This decrease must be explained in terms of the transformation of complex organics into intermediate species and carbon dioxide.^49^ Information provided by total organic carbon (TOC) is different than that provided by COD (and complementary), because its decay indicates only mineralization, that is, the transformation of organic carbon into the undesired carbon dioxide. As seen, there is a decay in the concentration of aromatic species, because they are transformed into carboxylates, increasing the concentration of total carboxylates contained in the system significantly. Thus, aromatic intermediates are formed during the process as the first stage of a process that continues with their oxidation to carboxylates and the further oxidation of these carboxylates to carbon dioxide (which, in turn, are transformed into bicarbonates or stripped as gases from the electrolyte). According to the literature, the electrolysis of organic matter in aqueous wastes proceeds primarily throughout the addition of hydroxyl groups to the pollutant molecules and the further oxidation of these groups breaking the raw and intermediate organic molecules into smaller ones, typically carboxylates, as schematized in eq 1.
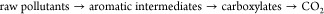
1

**Figure 3:**
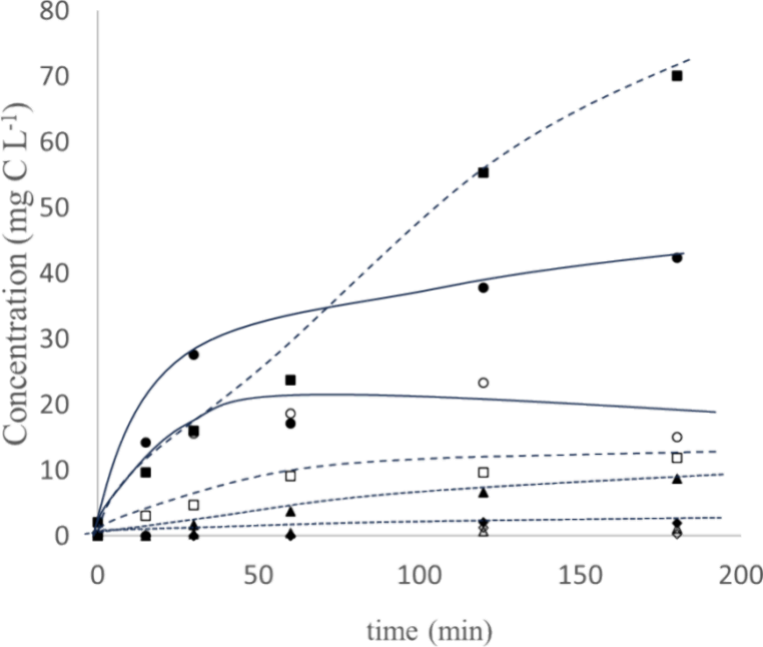
Details of the time course of the main carboxylate anions formed during the electrorefining of the cashew nut wastewater at 100 mA cm^–2^ (ELC) and 6 mA cm^–2^ (ESC) current densities. Full symbols: waste tank; empty symbols: recovery tank. Box solid, oxalate; triangle up solid, formate; tilted square solid, acetate; circle solid, others.

Total concentrations of carboxylates produced and transferred to the clean solution CRT tank in this test are shown in Figure 1b (separately from part a because of the very different scale), and as seen, they are important, both in the auxiliary reaction tank ART and in the carboxylates recovery tank CRT. More details about them are provided in Figure 3, which shows the changes in the concentration of specific carboxylates during the previous test in both compartments.

As seen, there are three main carboxylates detected (oxalate, formate, and acetate) in addition to a mixture of other carboxylates contained in much lower concentrations and totalized under the label “others”.

Among the primary carboxylates, oxalate seems to behave as the main product, observation that it is consistent to what has been found in the literature during the oxidation of most wastes using electrolytic technology.^50−53^ The primary intermediary in the oxidation of aromatic compounds is oxalate. This implies that the oxidation of carboxylates is not as rapid as the aromatic ring breakage.^54^ The more refractory oxidative character of oxalate facilitates their accumulation in the electrolysis tank, as compared to other carboxylates and salts, and this explains that it should be seen as a target compound in the development of this process. As seen, production is almost linear during the electrolysis with a production rate of 23.3 mg C h^–1^ in the case of oxalate and 2.90 mg C h^–1^ in the case of formate. Acetic acid is also an important organic species produced. It is important to note that the formation of acetic acid can also be explained in terms of the oxidation of the ethanol contained in the waste, because of its usage as a solvent during the treatment of the cashew nut wastes even when a complex water matrix should be considered (real biomass effluent). However, as experimentally confirmed, its production is less than 10% of the total ethanol used. This is important because acetate is not a typical intermediate in the degradation of aromatics, and in this work, it is measured at important concentrations. The production rate of this acid is also linear during the time of the test (0.34 mg C h^–1^). Within the mixture of other carboxylic acids, there is another carboxylate, which is worth highlighting, the propionic acid, but also other compounds that were identified by GC-MS. The anacardic acetate chromatographic peak was identified (304 m z^–1^) as well as the peaks of methyl (*E*)-9-octadecenoate (296 m z^–1^) and stearic acid methyl ester (298 m z^–1^) but in lower concentrations in the case of the latter compounds. It is important to note that these compounds also are considered as valuable compounds.

In the recovery tank, the TOC increases to 19.7 mg C L^–1^ during the duration of the test, following the transport of the formed carboxylates through the anionic exchange membrane. As well, the COD increases in 24.3 mg L^–1^ indicating that degradation in this tank should not be very important, and the key process happening in this zone is the transport and accumulation of carboxylates. In addition, opposite to carboxylates, negligible amounts of aromatic species are transported through the electrodialysis process from the reaction to the recovery tank and concentrations measured are really negligible (very low crossover). This can be considered as a very important outcome because the recovery solution can be ready for further processing as bricks in electrosynthesis, without the need of further purification. The main intermediate in this recovery tank is the oxalate, which it is explained by its higher concentration in the reaction tank. The other acids are also transported to the recovery tank following spitting ratios of 17.14, 11.43, 36.75, and 47.78%, respectively, for oxalate, formate, acetate, and the other carboxylates.

**Figure 4:**
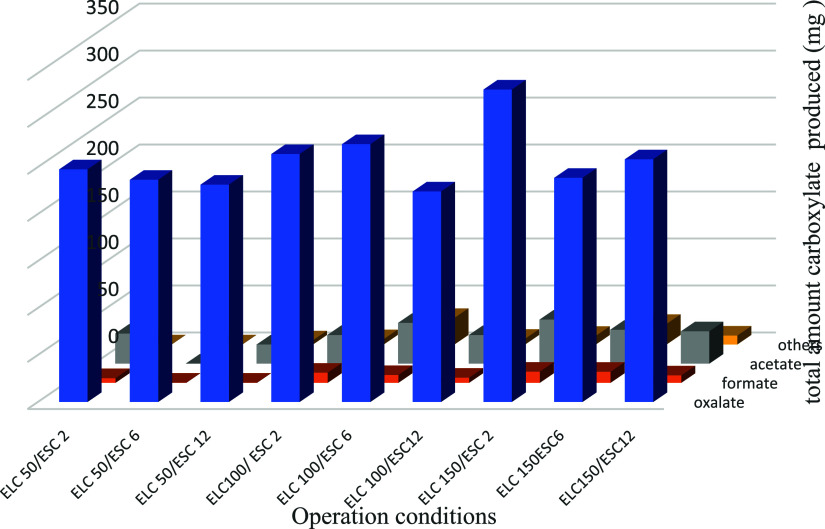
Total production of carboxylates after the application of 3 h of electrolysis at different operation current densities in the ELC and ESC cells. ELC(*j*)/ESC(*j*).

Results obtained in this test to show the performance of the technology were qualitatively reproduced when the electrolytic and electrodialytic current densities are changed (halving and doubling the value in each case, with the operation of nine different tests), and again, the same intermediates and similar time course of the concentrations were found. A more detailed description of the influence of these operation current densities is made in terms of the final products obtained after the application of the tests for an arbitrary period of 3 h. Thus, Figure 4 shows the effect of the current density exerted in the electrolyzer and electrodialyzer during the tests performed, on the production of carboxylates. As seen, within the ranges of current densities applied in the electrolysis and electrodialysis, oxalate is always the key carboxylate formed and there is an important influence of current densities applied in the electrolytic cell, since the production seems to be favored at large current densities, being the production of oxalate, formate, acetate, and other carboxylates increased by 14.97, 109.34, 62.43, and 90.52%, respectively, when operating at 150 with respect to operation at 50 mA cm^–2^. The lower impact on oxalate of the increase in the current density can be explained in terms of the refractory character of oxalate toward the mediated oxidation with hydroxyl radical, mechanism that it is known to be promoted over the direct electrolytic oxidation on the surface of the anode when operating at high current densities. Oppositely, formate and acetate are known to be efficiently degraded by these radicals formed in most wastewater treatment electrolytic processes and this is reflected in the very high improvements observed with the increase in the electrolytic operation current density.^34−36^

**Figure 5:**
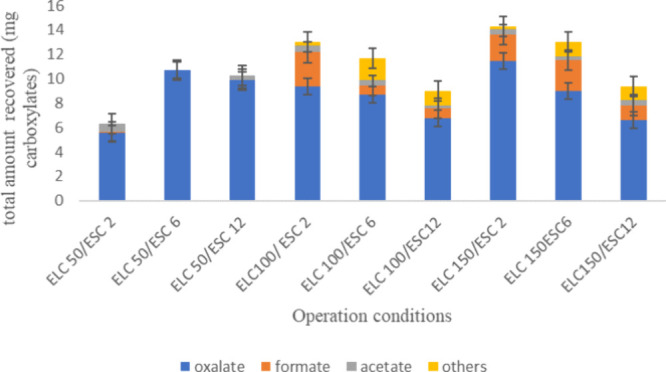
Total amounts of carboxylates accumulated in the CRT at different operation current densities in the ELC and ESC cells. ELC(*j*)/ESC(*j*).

Carboxylates concentrated in the CRT follow the same trend observed in the ART, as shown in Figure 5, which can be explained because the driving force in the purification of carboxylic acids is not only the cell voltage but, most importantly, the concentration in the ART in which carboxylates produced electrochemically are accumulated. In addition, the application of higher current densities in the electrodialysis seems to be negative, especially when the current applied in the electrolysis is also high. An explanation to this observation could be the partial mineralization of the carboxylic acids in the anode of the ESC cell when applying such current densities, which are close to those considered suitable for degradation.

**Figure 6:**
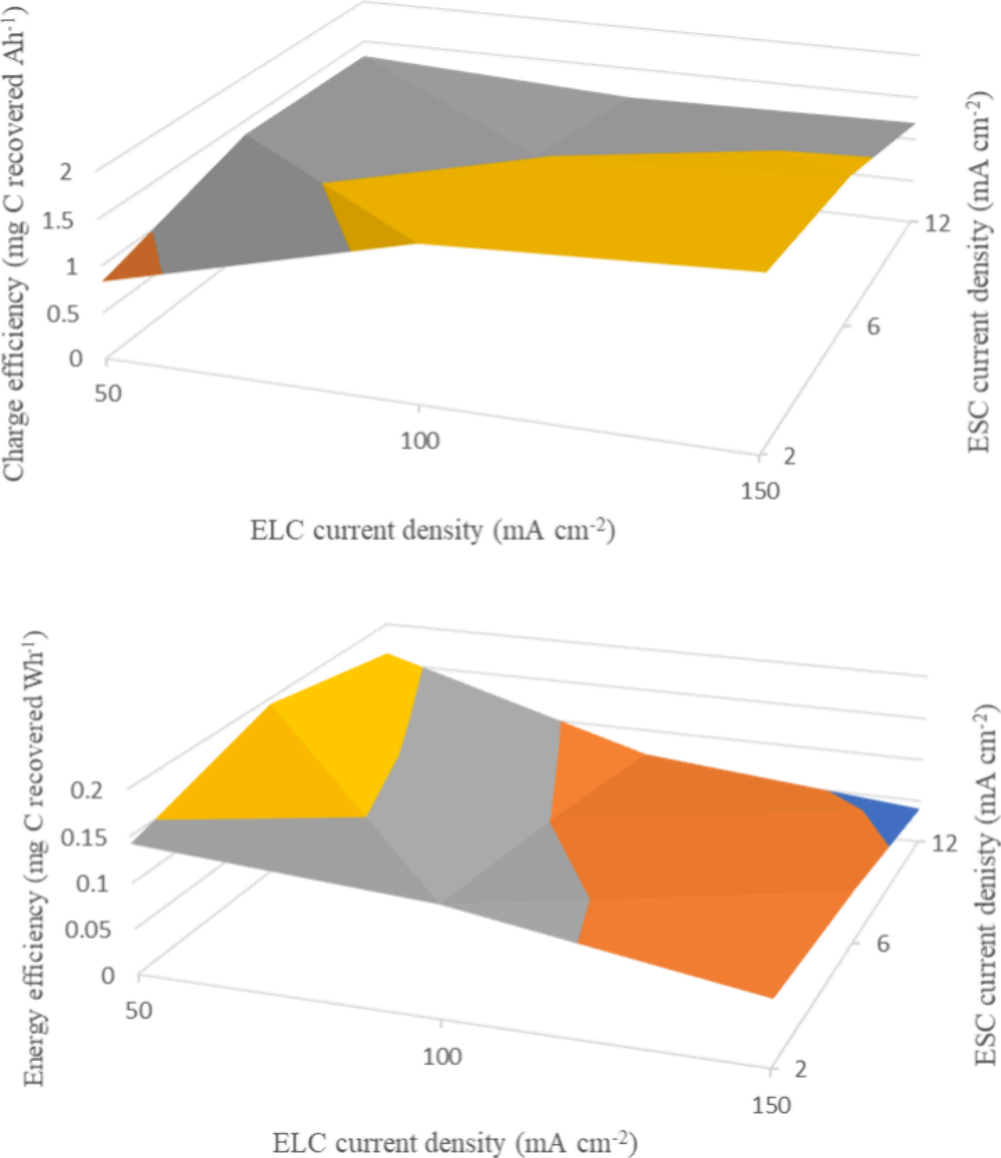
Effect of the operation current densities on the charge (above) and energy (below) efficiencies.

Finally in this work about a proof of concept, it is worth to evaluate efficiencies. Thus, Figure 6 shows the efficiencies (both Coulombic and energy) obtained during the different tests carried out. It can be seen that Coulombic efficiency ranges between 0.92 and 2.03 mg C (Ah)^−1^ while energy efficiencies range from 0.04 to 0.21 mg C (Wh)^−1^. The efficiencies are lower than the ones shown in Table 1, where no electroseparation was implemented, although the added value of the stream is much higher, because of its purity.

Values reached are not high, but even that, they are enough to demonstrate the feasibility of the process and the necessity to make a bet for their development. The effect of current densities on charge efficiency is not significant, although it indicates a positive effect of increasing the electrolysis current density and decreasing the electrodialysis current density for profit of the electrons flow. However, the effect of the energy efficiency is clearer because it is strongly influenced by the cell voltage, whose value increases with the operation current densities because of the higher ohmic losses and electrochemical rates. Because of that, from this efficiency criteria, it could be advisable to operate in the lower range of electrolytic current density. Anyhow, these results are still preliminary, and they only show the potentiality of this paradigmatic change of perspective of the treatment of real wastes. Other experimental conditions should be investigated such as the oxidative process by using a membrane, separating anodic and cathodic compartments and, consequently, avoiding reductive reactions. On the one hand, the implications of this additional mode could favor the deactivation of the anode surface, as already demonstrated when the treatment of the effluent was the main scope. Then, the oxidative conditions should be studied again. On the other hand, the use of different membranes in the electroseparation reactor could implicate an enhancement in the efficiency of the recovery, and the adjustment of the pH conditions could promote a “buffering state”, which favors the separation of carboxylates from carboxylic acids.

### Sustainability Analysis

Despite the low TRL of the study made, to start thinking of the development of a new business model concept for improving the environmental management of biosphere resources using the electrorefinery concept from the results obtained in this proof of concept with effluents from cashew nut production, it is necessary to assess the scope of this model, as well as to make an initial inventory of the environmental and social situation involved.

At this point, it is important to consider that Côte d’Ivoire, India, Tanzania, Benin, and Indonesia have the largest areas under cashew nut cultivation, accounting for 71% of the global area.^40^ Currently, most of the value added to cashew nuts harvested in drying, shelling, and removal of the second skin attached to the nut. The nuts are then sold in Europe and North America, where 60% of the nuts are roasted, salted, and packaged. Governments in the main producing countries, such as Côte d’Ivoire, want to increase local processing to increase the profitability and benefits of the crop in the country of origin, so multinational investment is increasing and processing centers are expanding.^55^

The development of cashew processing plants in the countries of origin should incorporate the concept of “refineries” (not only bio- but also electrorefinery), making use of the remaining biomass that is not marketed on a global scale, especially those resources that are needed by the agroindustrial sectors involved. This would reduce costs in the production chain, promote the autonomy of these sectors, and ensure that the environmental and social impact of industrial activity is as positive as possible. Thermal energy derived from diesel fuel used in cashew processing in currently installed plants accounts for about 90% of the energy cost of processing, with the drying and roasting stages consuming the most fuel.^56^ An alternative could be the use of residual biomass, as diesel engines can also run on cashew nut shell oil as fuel,^44^ if this oil is no to commercialized, and on the other hand, the integration of technology to carry out cogeneration, combustion, pyrolysis, or gasification of solid biomass by integrating CO_2_ capture technology to reduce carbon footprint. These biomass management strategies can be developed to meet the energy needs of the process itself or to serve as a feedstock for the region’s energy sector, reducing the cost and environmental impact of transporting low-value biomass fractions. In fact, the exergy study of a safflower biorefinery highlights the importance of this concept and concludes that the amount of agricultural waste generated is reduced but that 70% of the total irreversibility losses of the plant occur in the wastewater treatment unit. The irreversibility content quantified by exergy analysis is directly related not only to thermodynamic losses but also to economic losses/resource depletion.^3,57,58^ Therefore, the development of this new concept of organic electrorefinery would increase the efficiency of resource use by using these waste effluents as feedstock.

Life cycle assessment studies using electrochemical technology to treat pesticide-contaminated water conclude that more than 95% of the carbon and water footprint impact associated with electrochemical treatment is due to energy consumption, and this is very significantly reduced with the use of renewable energy.^4^ The facility had an electro-oxidation unit very similar to that of the facility in this study. For this reason, in order to estimate the carbon and water footprint of this organic electrorefinery proof-of-concept per milligram of carbon of the carboxylic acids obtained, the energy consumption of the energy mix of cashew nut processing countries such as India, Brazil, and Spain (the latter as a European country reference) was taken into account and compared with the same energy requirement provided by solar panels, biogas cogeneration, or wood chips (biomass that has similarities with cashew nut shells) trying to use LCA tools to shed light on how the electrorefinery concept may impact on two of the key sustainability outputs. Table 2 shows the carbon and water footprint impact categories per milligram of C produced. It can be determined that the impacts are reduced in those countries with a higher influence in their energy mix of the proportion of renewable energies, such as Brazil, due to its important percentage of hydroelectric energy in its energy mix. It is also observed that the use of renewable energies such as solar panels or biomass cogeneration in the carboxylic acid valorization stage is more sustainable alternatives. The carbon and water footprint of using cashew nutshell biomass can be reduced by 80–99.9% compared to the footprint of using grid electricity in the various processing countries.

**Table 2:** Carbon and Water Footprint of Energy Supply Required per Milligram of Carbon of Product

supply	**carbon footprint(g CO**_**2**_**eq/mg C)**	**water use**(L/mg C)
Spanish electric grid	2.89	0.76
Brazilian electric grid	1.61	0.90
Indian electric grid	9.91	1.49
solar powering	0.39	0.08
biogas cogeneration	1.84	0.23
wood cogeneration	0.31	0.03

In addition to these preliminary results, it is important to consider that this new concept of carboxylic acid synthesis is environmentally friendly, as it does not require the addition of chemical reagents, it does not have a high energy requirement, and organic oily residues from cashew nut processing and caustic water from cashew nut washing can be treated together. This also makes it interesting to compare this technology with industrial alternatives to produce oxalic acid, which are mainly based on the oxidation of different organics, such as ethylene glycol and olefins such as propene in the presence of nitric acid and catalysts such as vanadium oxide or using high temperatures (190 °C) and pressures (70 bar). Not least, the proposed treatment of this organic oily waste does not produce sludge or hazardous gaseous emissions and is carried out in closed equipment.^59^

A last point regarding improvements in sustainability is the potential use of oxalates as soil conditioners for cashew crops and/or for other crops in the countries of origin, as these compounds are known to improve the properties of soils and crops. Mineral weathering is facilitated by low-molecular-weight carboxylic acids.^60,61^ and currently there is ongoing work^62,63^ into the impact of the oxalate-carbonate pathway (OCP), which functions as an organic carbon sink. Therefore, the generation and application of low-molecular-weight carboxylic acids to soils and crops can form part of the development of strategies for atmospheric CO_2_ sequestration, imitating and accelerating the behavior of nature. This new concept of organic electrorefinery provides a sustainable alternative that could also increase the lifespan and yield of cashew plantations, which would increase the profitability of the crop, enhancing the circular economy of cashew production.

## Conclusions

From this work, the following conclusions can be drawn:

- Another perspective of the electrochemical treatment of wastes polluted with organic is feasible because it is possible to produce and separate carboxylates produced during the electrolytic degradation of a real wastewater produced in an agrifood industry and limit the emissions of carbon dioxide while providing a very interesting raw matter with many important applications
- Oxalate is the primary species accumulated because it is one of the most refractory intermediates produced in the degradation of organic pollutants contained in wastewater. A great variety of carboxylates are also detected although only formate, acetate, and propionate are detected at non-negligible concentrations.
- There is a negligible crossover of aromatic species into the recovery solution, which becomes an important advantage for further processing of the carboxylates solutions to valorize these species in terms of circular economy principles.
- Energy efficiencies in the range of 0.04–0.21 mg C-carboxylates Wh^1–^ and Coulombic efficiencies in the range of 0.92–2.03 mg C-carboxylates Wh^1–^ can be obtained in this preliminary work. Interesting to work at lower current densities to promote more sustainable processes by using more efficiently electric current minimizing ohmic loses.
- A comparative study of the carbon and water footprint of this proof of concept has been carried out by studying the influence of the type of energy supplied. A life cycle assessment indicated carbon dioxide and water footprints as low as 0.31 g CO_2_ mg^–1^ C and 30 mL H_2_O mg^–1^ C recovered in the products obtained, respectively.
- This technology is in the initial stages of the electrorefinery concept in organics, and there is still a long way for this one to be applied, but results obtained are promising and shed light on the new developments expected for a more sustainable electrochemical treatment of wastewater. For example, other compounds like anacardic acetate could be produced, concentrated, and separated because it is another high value-added product, which is a precursor for resins, coatings, and frictional materials.
- A sustainable alternative is to apply the resulting product, a solution of carboxylic acids, mainly oxalic acid, to soil crops to strengthen plants and mineralize atmospheric CO_2_ in soils.

## Notations

pH: pH (−)
p*K*_a_: p*K*_a_ (−)
**Abbreviations**
ART: auxiliary reaction tank
BDD: boron-doped diamond
COD: chemical oxygen demand
CO_2_: carbon dioxide
CRT: carboxylate recovery tank
CNSL: cashew nutshell liquid
ELC: electrolysis cell
ESC: electroseparation cell
GC-MS: gas chromatography coupled to mass spectrometry
HPLC: high-performance liquid chromatography
MMO: mixed metal oxide
TOC: total organic carbon
TRL: technology readiness level
